# The cyanobacterial cell division factor Ftn6 contains an N-terminal DnaD-like domain

**DOI:** 10.1186/1472-6807-9-54

**Published:** 2009-08-21

**Authors:** Martial Marbouty, Cyril Saguez, Franck Chauvat

**Affiliations:** 1 CEA, iBiTec-S, SBIGeM, LBI, Bat 142 CEA-Saclay, F-91191 Gif sur Yvette CEDEX, France

## Abstract

**Background:**

DNA replication and cell cycle as well as their relationship have been extensively studied in the two model organisms *E. coli *and *B. subtilis*. By contrast, little is known about these processes in cyanobacteria, even though they are crucial to the biosphere, in utilizing solar energy to renew the oxygenic atmosphere and in producing the biomass for the food chain. Recent studies have allowed the identification of several cell division factors that are specifics to cyanobacteria. Among them, Ftn6 has been proposed to function in the recruitment of the crucial FtsZ proteins to the septum or the subsequent Z-ring assembly and possibly in chromosome segregation.

**Results:**

In this study, we identified an as yet undescribed domain located in the conserved N-terminal region of Ftn6. This 77 amino-acids-long domain, designated here as FND (Ftn6 N-Terminal Domain), exhibits striking sequence and structural similarities with the DNA-interacting module, listed in the PFAM database as the DnaD-like domain (pfam04271). We took advantage of the sequence similarities between FND and the DnaD-like domains to construct a homology 3D-model of the Ftn6 FND domain from the model cyanobacterium *Synechocystis *PCC6803. Mapping of the conserved residues exposed onto the FND surface allowed us to identify a highly conserved area that could be engaged in Ftn6-specific interactions.

**Conclusion:**

Overall, similarities between FND and DnaD-like domains as well as previously reported observations on Ftn6 suggest that FND may function as a DNA-interacting module thereby providing an as yet missing link between DNA replication and cell division in cyanobacteria. Consistently, we also showed that Ftn6 is involved in tolerance to DNA damages generated by UV rays.

## Background

DNA replication and cell division are probably the most fundamental processes in the cell life cycle. Both proceed through a remarkably conserved general mechanism and are inextricably intertwined to each others and to the cell metabolism [1].

The DNA replication cycle can be divided into three distinct stages; initiation, elongation, and termination. Replication is initiated by the highly conserved AAA+ superfamily ATPase-member DnaA that binds the *oriC*, inducing DNA strand melting [2-4]. In *E. coli*, DnaA also interacts with the ring helicase DnaB and directs the loading of DnaB/DnaC onto the single stranded DNA (ssDNA) region. After binding of DnaB on the ssDNA region of the *oriC*, DnaC is released in an ATPase dependent manner. Then, DnaB recruits the DnaG primase and DNA polymerase III to form the replication fork [4]. In *B. subtilis*, two additional essential proteins, called DnaB (be aware of the confusing nomenclature between the *E. coli *and *B. subtilis*) and DnaD, are engaged in entry of the ring helicase at *oriC*. DnaB could function as a membrane anchoring factor for the replication initiation machinery [5] or, together with DnaI, the functional homolog of *E. coli *DnaC [6], in the recruitment of the ring helicase [7]. DnaD interacts with both DnaA and DnaB [8,9]. It exhibits DNA remodelling activity, enhancing partial melting of the DNA strands, and could, therefore, function in early steps of replication such as initiating the recruitment of the ring helicase [9-15]. During elongation, the replication forks constituted at the *oriC *travel in opposite directions to achieve replication of the entire chromosome. When the replication forks reach the terminus, terC, the replication complexes are dismantled in a process involving specific termination factors [16].

The earliest event in bacterial cytokinesis is the definition of the future cell division site. This occurs through the dynamic assembly/disassembly of the Z-ring structure resulting from the self-polymerization of the ubiquitous tubulin-like protein FtsZ [17,18]. Placement of the Z-ring is mainly dependent on the Min system both in *E. coli *and in *B. subtilis *[17,18]. Once assembled, the Z-ring is believed to serve as a scaffold for recruitment of the cell division machinery to activate septation and physical separation of the daughter cells. In contrast to the model organisms *E. coli *and in *B. subtilis*, the molecular basis of cell division has not been as well studied in cyanobacteria. Nevertheless, studies with the two unicellular cyanobacteria *Synechocystis *PCC6803 and *Synechococcus *PCC7942 and the filamentous *Anabaena *PCC7120, have allowed the identification and the characterisation of clear Fts and Min orthologs as well as ZipN/Ftn2 and Ftn6, two cell division factors restricted to cyanobacteria [19-21]. Although *ftn6 *deletion leads to cell division defects, resulting in cells dramatically elongated in *Synechococcus *PCC7942 or enlarged in *Anabaena *PCC7120 [19,21], the molecular function of Ftn6 remains unclear. Nevertheless, recent data suggest an involvement of Ftn6 in recruitment of FtsZ proteins to the septum or subsequent Z-ring assembly, as cells deleted for *ftn6 *do not exhibit condensed Z-rings, but rather diffuse localization of FtsZ [21].

Based on sequence and structure analyses, we here propose that the cyanobacterial-specific cell division factor Ftn6 contains a not hitherto described N-terminal domain related to the DnaD-like domain found in the DnaD chromosomal replication protein family. Identification of the Ftn6 N-terminal Domain, we termed FND (Ftn6 N-terminal Domain), opens up very interesting perspectives about Ftn6 function in cell division and possibly in chromosome segregation as well as on their necessary interplay. Consistently, we also showed that Ftn6-depleted cells are sensitive to DNA damages generated by UV rays.

## Results and discussion

### Ftn6 orthologs contain a conserved N-terminus domain (FND)

**Figure 1:**
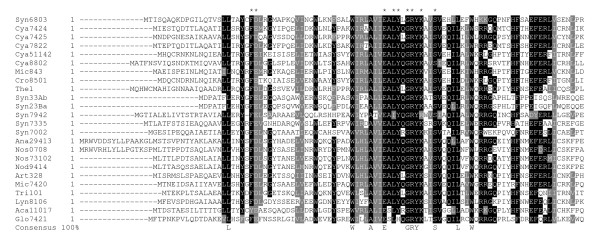
**Multiple sequence alignment of the N-terminal domain of Ftn6 orthologs**. BoxShade representation of the multiple alignment of the N-terminal domain of Ftn6 orthologs built with ClustalW2. Organisms, accession numbers and characteristics of the Ftn6 sequences shown in the alignment are given in the additional file 1. The starting residues are reported at the front of the corresponding sequence. Amino acids identical or similar in 70% of the sequences are shaded by black or grey background, respectively. The consensus in 100% of the sequences is indicated below the alignment. Stars point out the conserved residues exposed onto the surface of the FND structure (see text).

To identify putative Ftn6 motifs amenable for functional analysis, we performed database searches using the *Synechocystis *PCC6803 Ftn6 protein sequence (Syn6803) as query. BLAST search of the NCBI-database allowed the identification of 27 Ftn6 orthologs, all belonging to the cyanobacteria phylum. No ortholog was found in plastids or other prokaryotes. Interestingly, Ftn6 orthologs were found in all Nostocales (5 out of 5), Oscillatoriales (4/4) and Gloeobacterales (1/1), in some Chroococcales (17/29), but not in Prochlorales (0/13) even in those whose genome is fully sequenced. This finding, along with the viability of *ftn6*-depleted mutants [19,21], suggests that other cell division factors functionally overlap with Ftn6 in cytokinesis. Alignment of all Ftn6 amino-acids sequences identified by BLAST (Additional file 1) revealed a single conserved region encompassed within the first 90 first amino-acids of Syn6803 Ftn6 (Figure 1). This 77 amino-acids-long domain (L_18 _to L_94 _in Syn6803 Ftn6), termed here FND for Ftn6 N-terminal Domain, is bipartite with the first 28 amino-acids (L_18 _to L_45_) poorly conserved and the 49 remaining ones (W_46 _to L_94_) characterized by the W-X_3_-A-X_2_-E-X_4_-G-R-Y-X_3_-S-X_4_-L-X_2_-W consensus (Figure 1). The high degree of conservation of FND in Ftn6 orthologs suggests that this domain plays an important part of the function(s) of Ftn6 in cell division.

### FND is related to the DnaD-like domain

**Figure 2:**
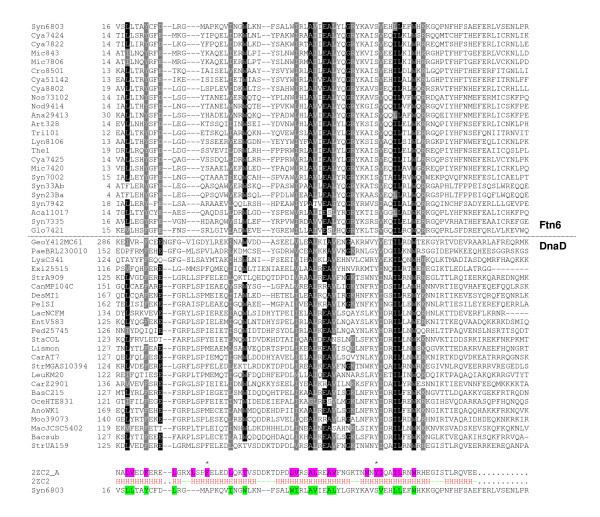
**Multiple sequence alignment of FND and DnaD-like domains**. Sequences were aligned using ClustalW2 and shaded with BoxShade. The starting residues are reported at the front of the corresponding sequence. Amino acids identical or similar in 70% of the sequences are shaded by black or grey background, respectively. Organisms, accession numbers and characteristics of DnaD-like and FND sequences shown in the alignment are given in the additional files 1 and 4. 2D-structure of *Streptococcus mutans *DnaD-like domain (PDB: 2ZC2) is shown at the bottom of the alignment. Helices and loops are represented in red and green respectively. Hydrophobic positions conserved in DnaD (Denoted by red dotes in the additional file 2) are shaded by pink background and their equivalent in FND (Figure 1) shaded by light gren background. Stars indicate the two conserved hydrophobic positions that are not buried in the *Streptococcus mutans *DnaD-like domain.

Interrogation of protein domain databases did not allow identification of any FND-related domain (data not shown). Conversely, PSI-BLAST searches of the NCBI non-redundant database using the Syn6803 FND sequence as query identified significant hits for the alignment of this sequence with its orthologs and, interestingly, with members of the DnaD protein family. DnaD sequences that were reported from the 3th PSI-BLAST iteration share low, typically less than 20% identity, but significant similarities with Ftn6 orthologs (Figure 2). For instance, the Psi-BLAST returned alignments with highly significant E-values between Syn6803 FND and several members of the DnaD-like family (*Geobacillus sp.*, GenBank:ZP_03559496, E-value 8e-15; *Lactobacillus hilgardii*, GenBank:ZP_03954546, E-value 1e-13; *Clostridium beijerinckii*, GenBank:YP_001308753, E-value 1e-10; *Streptococcus mutans*, GenBank: 2ZC2_A, E-value 4e-08 or *Bacillus subtilis*, GenBank:ABN10247, E-value 3e-08;...). DnaD consists of two domains with distinct biochemical properties [14]. The N-terminal Domain is involved in the oligomerization of the protein and interactions with DnaA, while the C-terminus, listed in the PFAM database as the DnaD-like domain (pfam04271), binds DNA [8,14]. Very interestingly, the sequence similarity we observed between the Ftn6 orthologs and the DnaD family is restricted to the DnaD-like domain (Figure 2), suggesting a DNA-binding activity for FND.

### Modelling FND

The belonging of FND to the DnaD-like domain family was further supported by fold recognition techniques. Indeed, submission of each FND domains to the PHYRE server constantly returned the two DnaD structures currently deposited in PDB as best hits, i.e. the DnaD-like domain of the replication proteins from *Streptococcus mutans *(PDB: 2ZC2) and from *Enterococcus faecalis *(PDB: 2I5U). DnaD-like domains reported from the PHYRE search share low but noticeable similarities with all FND tested (data not shown), suggesting structural similarities between FND and the DnaD-like domain. Then, *Streptococcus mutans *DnaD-like domain has been included in the alignment shown in Figure 2. As expected for proteins sharing a low level of sequence identity, we noticed that the nature of the hydrophobic residues conserved in DnaD-like domains (noted by red dots below the WebLogo profile [22] shown in the additional file 2 and shaded in pink background in Figure 2) was not preserved in FND (shaded by light green background in Figure 2). By contrast, their positions were highly conserved. The high degree of conservation (80%; 16 aminoacids residues out of 20; compare positions shaded in pink and green in the bottom of Figure 2) of the hydrophobic pattern between FND and DnaD-like domain strongly argues in favour of a similar fold. This is particularly evident for the helices 3 and 4, in which the hydrophobic pattern is not only conserved in position (83%; 10 out of 12), but is also highly similar, particularly the two alanines of the third helix (A_50 _and A_54 _in Syn6803 FND) and the leucine and the tyrosine (L_69 _and W_72 _respectively) at the extreme C-terminus of the fourth helix (Figure 2).

**Figure 3:**
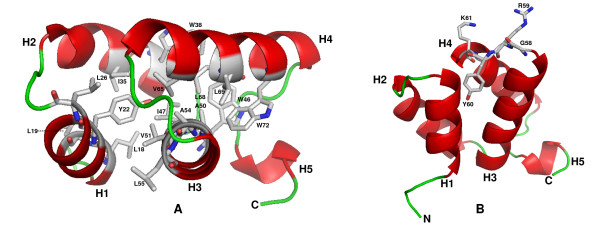
**Modelling FND**. (A) Pymol representation *Synechocystis *PCC6803 FND modelled with the MODELLER 9v6 program. Helices and loops are represented in red and green respectively. Hydrophobic positions of Syn6803 FND conserved in the DnaD-like domain (Figure 3A and additional file 2) are shown. (B) The highly conserved G_58_R_59_Y_60 _[K/R]_61 _motif localizes in a loop between the helices 3 and 4.

Based on these results and the alignment shown in Figure 2, we constructed a homology 3D-model of Syn6803 FND with MODELLER [23] Normalized DOPE z-score: -0.533). Overall model quality assessed by ProSA-Web returned a Z-Score of -4.12, which is in agreement with the Z-Scores of all experimentally determined chains currently deposited in the PDB database. Most of the hydrophobic positions of FND conserved in the DnaD-like domain (Figure 2 and additional file 2) are buried within the structure (Figure 3A), emphasizing their importance for structure stability and/or folding. Note that two hydrophobic positions conserved in DnaD-like domains (noted by a star in the Figure 2), but missing in FNDs, are not buried in the DnaD-like domains structure (data not shown) and, hence, are probably not required for the folding of this domain. The highly conserved G_58_R_59_Y_60 _[K/R]_61 _motif that is specific for FNDs (Figures 1 and 2), localizes in a loop between the helices H3 and H4 (Figures 1 and 3B). The tyrosine is buried within the structure, whereas the glycine and the two basic amino-acids are exposed on the surface of FND. Consurf-based mapping of the evolutionarily conserved residues exposed on the surface of the Syn6803 FND domain [24] (Additional file 3) show that G_58_, R_59 _and K_61 _residues cluster with F_24 _and D_25 _from the first helix, E_53_, L_55 _and Y_56 _from the third helix and S_64 _from the fourth helix. All these residues are either strictly or functionally conserved (Figure 1) and hence, could be engaged in Ftn6-specific interactions.

### Functional prediction for Ftn6

So far, DnaD-like proteins have only been found in some low G+C content gram positive bacteria and their associated phages [14], where they exhibit pleiotropic functions all related with DNA metabolism. For instance, DnaD was shown to be involved in initiation of chromosome and plasmid replication [25,26], sporulation [27], DNA repair [28] and recombination [29]. Furthermore, the DnaD-related protein from the thermophilic bacteriophage GBSV1 exhibits an unspecific nuclease activity [30]. The exact function of the DnaD-like domain in these processes remains unclear, but the DnaD-like domain from *B. subtilis *was found to exhibit DNA-binding and DNA-remodelling activities [11-15]. Altogether, these data strongly suggest that the DnaD-like domain does not define a common structural fold occurring in functionally unrelated proteins, but rather that the DnaD-like domain-containing proteins, including Ftn6, share common functions involving DNA.

What is the function of Ftn6? FND could suggest a function in DNA replication for Ftn6. However, this hypothesis is unlikely as *Ftn6*-depleted mutants do not appear to affect chromosome replication and do not produce anucleate cells [21]. Furthermore, the N-terminal extension in DnaD proteins, which interacts with DnaA [8], is missing in Ftn6 orthologs (data not shown). Alternatively, Ftn6 could function in the cross talk between chromosome replication and cell division, a fundamental biological process not yet investigated in cyanobacteria. In most bacteria, both processes are intimately co-ordinated, as formation and placement of the future division septum is regulated by nucleoid occlusion and only occurs after replication of a significant portion of the chromosome [1]. By contrast, Z-ring can assemble at nucleoid-occupied sites and nucleoid separation occurs during Z-ring constriction in cyanobacteria [21]. This lack of nucleoid occlusion supposes an efficient mechanism to segregate chromosome trapped at the midcell during Z-ring constriction. It has recently been proposed that Ftn6 could be involved in chromosome segregation in *Synechococcus *PCC7942 [31]. How Ftn6 is functionally connected to chromosome segregation remains unknown. Nevertheless, identification of the putative DNA-binding domain, FND, strongly supports the involvement of Ftn6 in this pathway and its interplay with cell division.

**Figure 4:**
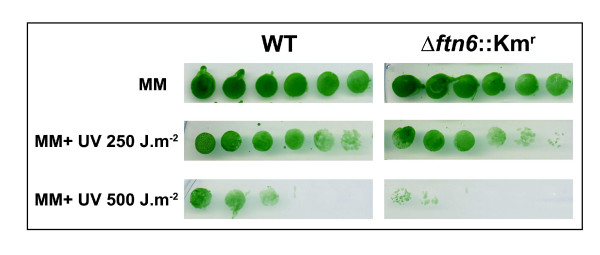
**Partial depletion of Ftn6 causes sensivity to UV**. WT and Ftn6-depleted cells (*Δftn6*::Km^r^) were grown to 0.5 OD_580 _and spotted in 4-fold serial dilutions onto MM plates. Then, the plates were exposed to 0 (control), 250 or 500 J.m^-2 ^UV rays.

To test this hypothesis, we reasoned that defective chromosomal segregation should generates DNA damages thereby increasing the sensitivity of the cells to DNA damaging agents. As expected, we found that the Ftn6-depleted mutant we recently constructed [32] is significantly more sensitive to UV rays than the wild-type strain (Figure 4). Together, our findings strengthen both the functional relationship between DnaD-like domain-containing proteins and DNA metabolism, and the potential function of Ftn6 at the interface between DNA replication and cell division.

## Conclusion

Although depletion of Ftn6 leads to cell division defects, the molecular function of this cynobacterial-specific divisome component remained unclear. Sequence alignment of Ftn6 orthologs beforehand identified by BLAST allowed us to uncover a new conserved domain localized within the N-terminus of the proteins. Combining several approaches, we then shown that this domain, designated here as FND, exhibits sequence and structure similarities with the DnaD-like domains found in several factors involved in DNA metabolism. The structure similarities between FND and DnaD-like domains together with the sensitivity of the Ftn6-depleted mutant to UV rays, led us to propose that Ftn6 is functionally linked to DNA metabolism, possibly playing a role at the interface between DNA replication and cell division. Whether this function involves or not other cell division factors and what is (are) the DNA target(s) of Ftn6 remain to be determined.

## Methods

### *In silico *methods

Databases search of Ftn6 and DnaD domain-containing proteins were performed using BLAST (e < 10-4) [33,34] and PsiBLAST [34,35] algorithms. Multiple sequence alignment of the DnaD-like-containing proteins or/and Ftn6 orthologs were generated using ClustalW2 [36,37] (Matrix: BLOSUM, Gap penality: 10 and penality for Gap extension: 0,1), and visualized with Boxshade [38]. Further details are given in the relevant figure legends and in the additional Files 1 and 4. Fold recognition was performed with PHYRE [39,40]. 3D-structure of Syn6803 FND was modelled using the MODELLER 9v6 program [23] and visualized with Pymol [41]. Briefly, 10 models of Syn6803 FND were first built based on the alignment shown in Figure 2. All 10 models were then evaluated with DOPE from the MODELLER package and the best chosen as final model. The overall model quality was additionally validated with ProSA-Web [42,43].

### UV-sensitivity tests

WT *Synechocystis *PCC6803 and its derivative *ftn6Δ::Km*^r^*/FTN6+ *[32] were grown as described [44]. Cells were then 4-fold serially diluted in MM medium and then spotted onto MM plates. Finally, the plates were or not exposed to either 250 or 500 J.m^-2 ^UV radiation and incubated 7 days at 30°C under the above described light conditions.

## Authors' contributions

MM and CS collected the data, participated in the sequences alignments and the modelling of the 3D-structures. FC and CS conceived the study, carried out the analysis of the data and drafted the manuscript. FC coordinated the study. All authors read and approved the final manuscript.

## Supplementary Material

Additional file 1 **Description of the Ftn6 sequences identified by BLAST**. The table reports the organisms, the Genbank accession numbers and the length of the Ftn6 sequences identified by BLAST and shown in the Figures 1 and 2. Click here for file

Additional file 2 **LOGO profile of the DnaD-like domains**. The LOGO profile was generated from the ClustalW [36] alignment of 82 randomly chosen non redundant DnaD-like sequences (data not shown) using WebLogo [22]. The red dots at the bottom of the alignment represent the hydrophobic positions conserved in the DnaD-like domain family. The 3D-structure shown at the top of LOGO profile corresponds to the DnaD-like domain of the replication proteins from *Streptococcus mutans *(PDB: 2ZC2). Click here for file

Additional file 3 **Surface amino-acid conservation of the FND domain**. The surface amino-acid conservation of the FND domain was calculated with Consurf [24] using the alignment shown in Figure 1. The colour-code shown at the bottom of the structure indicates the residues conservation. Briefly, residues are coloured from purple (highly conserved) to blue (non-conserved) depending on their respective conservation. Stars indicate strictly conserved amino-acids. The graphic was generated with Pymol. Click here for file

Additional file 4 **Description of the DnaD sequences shown in the Figure **2. The table reports the organisms, the Genbank accession numbers and the length of the DnaD sequences used to generate the alignment shown in the Figure 2. Click here for file
