# Supplementary material for: The cyanobacterial cell division factor Ftn6 contains an N-terminal DnaD-like domain
Source: BMC Struct Biol. 2009 Aug 21;9:54. doi: 10.1186/1472-6807-9-54 (PMC2736966; doi:10.1186/1472-6807-9-54)
Supplement: Additional file 1 — Description of the Ftn6 sequences identified by BLAST. The table reports the organisms, the Genbank accession numbers and the length of the Ftn6 sequences identified by BLAST and shown in the Figures 1 and 2. [file 1472-6807-9-54-S1.pdf]

Sequences name	Organisms	Orders	Accession numbers	Proteins length	Notes
**Syn6803**	Synechocystis PCC 6803	Chroococcales	NP_440426	214	
**Cya7424**	Cyanothece sp. PCC 7424	Chroococcales	YP_002376006	297	
**Cya7425**	Cyanothece sp. PCC 7425	Chroococcales	YP_002482808	214	
**Cya7822**	Cyanothece sp. PCC 7822	Chroococcales	ZP_03155552	296	
**Cya51142**	Cyanothece sp. ATCC 51142	Chroococcales	YP_001805611	224	
**Cya8802**	Cyanothece sp. PCC 8802	Chroococcales	ZP_03142194	228	Identical to Cyanothece sp. PCC 8801 Ftn6 (AC: YP_002371108), 228aa
**Mic843**	Microcystis aeruginosa NIES-843	Chroococcales	YP_001660954	227	83% identical to Microcystis aeruginosa PCC 7806 Ftn6 (AC: CAO87789), 219aa
**Cro8501**	Crocosphaera watsonii WH 8501	Chroococcales	ZP_00517619	224	
**The1**	Thermosynechococcus elongatus BP-1	Chroococcales	NP_680857	155	
**Syn33Ab**	Synechococcus sp. JA-3-3Ab	Chroococcales	YP_474634	183	
**Syn23Ba**	Synechococcus sp. JA-2-3B'a(2-13)	Chroococcales	YP_478788	175	
**Syn7942**	Synechococcus elongatus PCC 7942	Chroococcales	AAL16072	152	99% identical to Synechococcus elongatus PCC 6301 Ftn6 (AC: YP_173094), 143aa
**Syn7335**	Synechococcus sp. PCC 7335	Chroococcales	YP_002711975	220	
**Syn7002**	Synechococcus sp. PCC 7002	Chroococcales	YP_001734859	152	
**Ana29413**	Anabaena variabilis ATCC 29413	Nostocales	YP_324721	228	95% identical to Nostoc sp. PCC 7120 Ftn6 (YP_324721), 227aa
**Nos0708**	'Nostoc azollae' 0708	Nostocales	ZP_03763738	244	
**Nos73102**	Nostoc punctiforme PCC 73102	Nostocales	YP_001864302	260	
**Nod9414**	Nodularia spumigena CCY 9414	Nostocales	ZP_01629727	232	
**Art328**	Arthrospira maxima CS-328	Oscillatoriales	ZP_03272428	162	
**Mic7420**	Microcoleus chthonoplastes PCC 7420	Oscillatoriales	YP_002618935	221	
**Tri101**	Trichodesmium erythraeum IMS101	Oscillatoriales	YP_722793	187	
**Lyn8106**	Lyngbya sp. PCC 8106	Oscillatoriales	ZP_01623531	178	
**Aca11017**	Acaryochloris marina MBIC11017	unclassified	YP_001519480	245	
**Glo7421**	Gloeobacter violaceus PCC7421	Gloeobacteria	NP_926672	140	
