# Supplementary figures and images for: The cyanobacterial cell division factor Ftn6 contains an N-terminal DnaD-like domain
Source: BMC Struct Biol. 2009 Aug 21;9:54. doi: 10.1186/1472-6807-9-54 (PMC2736966; doi:10.1186/1472-6807-9-54)

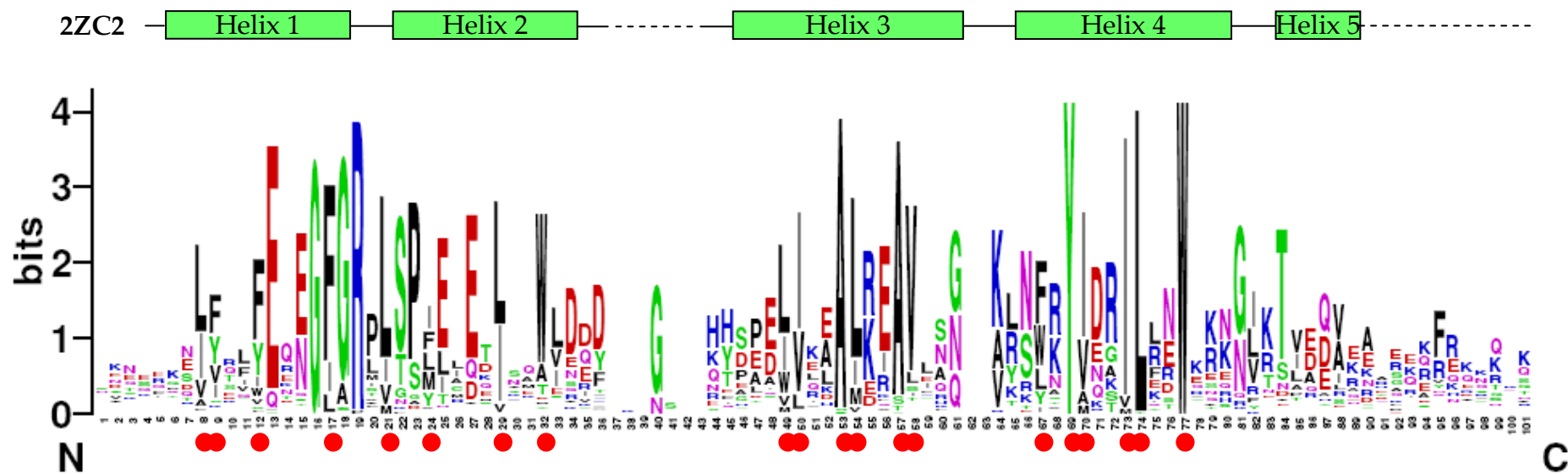

Supplement: Additional file 2 — LOGO profile of the DnaD-like domains. The LOGO profile was generated from the ClustalW [36] alignment of 82 randomly chosen non redundant DnaD-like sequences (data not shown) using WebLogo [22]. The red dots at the bottom of the alignment represent the hydrophobic positions conserved in the DnaD-like domain family. The 3D-structure shown at the top of LOGO profile corresponds to the DnaD-like domain of the replication proteins from Streptococcus mutans (PDB: 2ZC2). [file 1472-6807-9-54-S2.pdf]

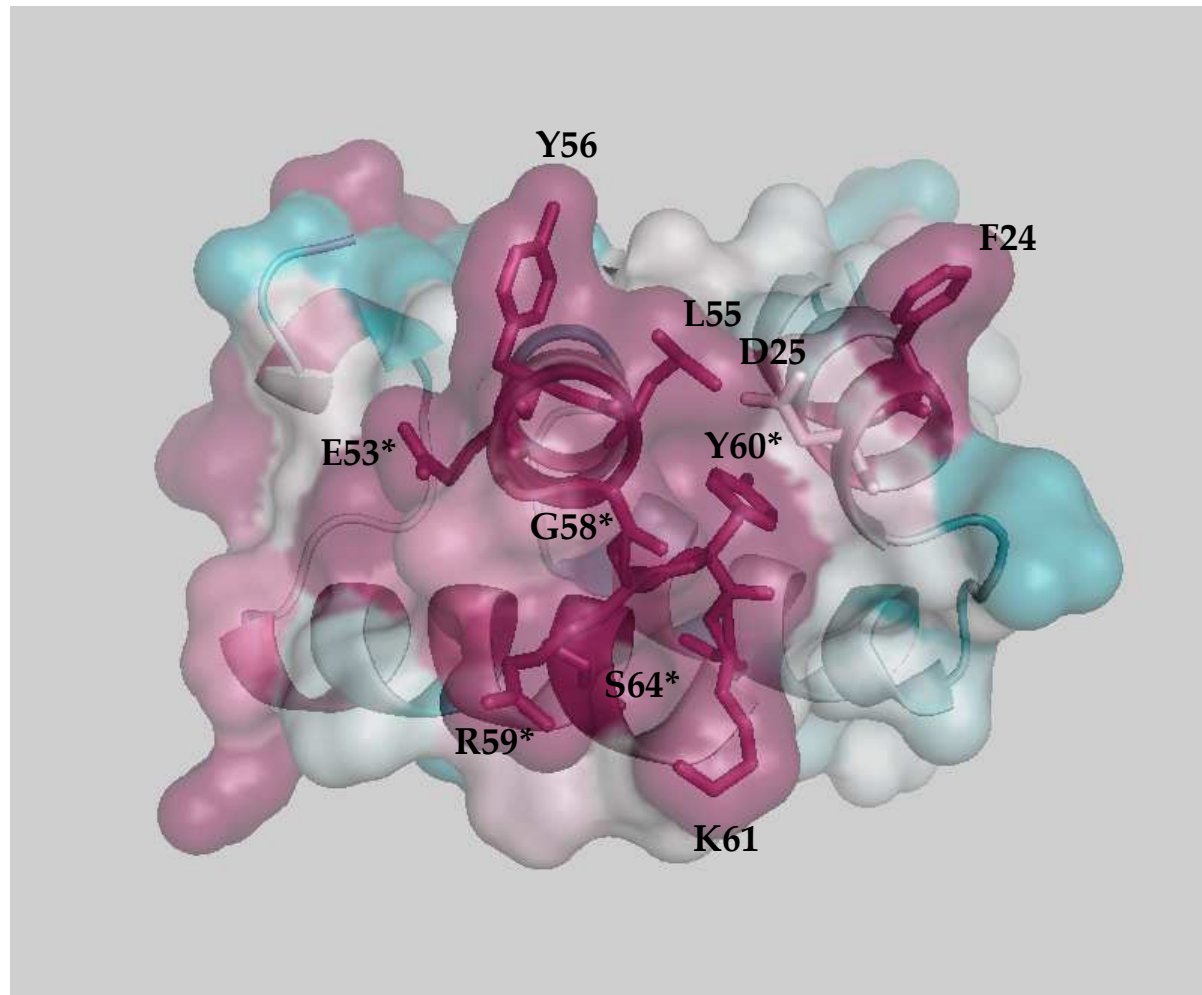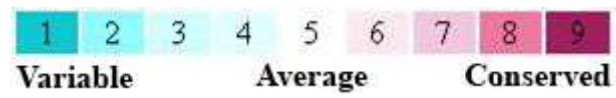

Supplement: Additional file 3 — Surface amino-acid conservation of the FND domain. The surface amino-acid conservation of the FND domain was calculated with Consurf [24] using the alignment shown in Figure 1. The colour-code shown at the bottom of the structure indicates the residues conservation. Briefly, residues are coloured from purple (highly conserved) to blue (non-conserved) depending on their respective conservation. Stars indicate strictly conserved amino-acids. The graphic was generated with Pymol. [file 1472-6807-9-54-S3.pdf]
