# Supplementary material for: The cyanobacterial cell division factor Ftn6 contains an N-terminal DnaD-like domain
Source: BMC Struct Biol. 2009 Aug 21;9:54. doi: 10.1186/1472-6807-9-54 (PMC2736966; doi:10.1186/1472-6807-9-54)
Supplement: Additional file 4 — Description of the DnaD sequences shown in the Figure 2. The table reports the organisms, the Genbank accession numbers and the length of the DnaD sequences used to generate the alignment shown in the Figure 2. [file 1472-6807-9-54-S4.pdf]

Sequences name	Organisms	Phyllum	Accession numbers	Proteins length	Notes
GeoY412MC61	Geobacillus sp. Y412MC61	Firmicutes	ZP_03559496	430	
PaeBRL230010	Paenibacillus larvae subsp. larvae BRL-230010	Firmicutes	ZP_02328899	260	
LysC341	Lysinibacillus sphaericus C3-41	Firmicutes	YP_001698185	254	
Exi25515	Exiguobacterium sibiricum 255-15	Firmicutes	YP_001814241	192	
StrA909	Streptococcus agalactiae A909	Firmicutes	YP_329904	226	
CanMP104C	Candidatus Desulforudis audaxviator MP104C	Firmicutes	YP_001717037	260	
DesMI1	Desulfotomaculum reducens MI-1	Firmicutes	YP_001113445	280	
PelSI	Pelotomaculum thermopropionicum SI	Firmicutes	YP_001211559	280	
LacNCFM	Lactobacillus acidophilus NCFM	Firmicutes	YP_194031	214	
EntV583	Enterococcus faecalis V583	Firmicutes	NP_814884	238	
Ped25745	Pediococcus pentosaceus ATCC 25745	Firmicutes	YP_804430	234	
StaCOL	Staphylococcus aureus subsp. aureus COL	Firmicutes	YP_186337	228	
Lismon	Listeria monocytogenes EGD-e	Firmicutes	NP_465419	239	
CarAT7	Carnobacterium sp. AT7	Firmicutes	ZP_02185493	238	
StrMGAS10394	Streptococcus pyogenes MGAS10394	Firmicutes	YP_060073	227	
LeuKM20	Leuconostoc citreum KM20	Firmicutes	YP_001727927	212	
CarZ2901	Carboxydothermus hydrogenoformans Z-2901	Firmicutes	YP_360073	245	
BasC215	Bacillus halodurans C-125	Firmicutes	NP_242563	233	
OceHTE831	Oceanobacillus iheyensis HTE831	Firmicutes	NP_692679	225	
AnoWK1	Anoxybacillus flavithermus WK1	Firmicutes	YP_002315496	272	
Moo39073	Moorella thermoacetica ATCC 39073	Firmicutes	YP_430042	249	
MacJCSC5402	Macrococcus caseolyticus JCSC5402	Firmicutes	YP_002560504	224	
Bacsub	Bacillus subtilis	Firmicutes	ABN10251	232	
StrUA159	Streptococcus mutans UA159	Firmicutes	NP_721817	231	DnaD Structure ID: 2ZC2
